# Sirolimus potentiated angioedema: A case report and review of the literature

**DOI:** 10.1515/med-2023-0884

**Published:** 2024-01-09

**Authors:** Hadi Beaini, Carol Bjorkman, Kelly Johnson, Faris G. Araj

**Affiliations:** Division of Cardiology, Department of Internal Medicine, The University of Texas Southwestern Medical Center, Dallas, United States; Division of Cardiology, Department of Internal Medicine, The University of Texas Southwestern Medical Center, Professional Office Bldg. 2 Suite 600, 5939 Harry Hines Blvd. Dallas, TX, 75390-9252, United States

**Keywords:** adverse drug reactions, heart transplantation, sirolimus, angioedema

## Abstract

**Introduction:**

In the realm of organ transplantation, particularly heart transplantation, angioedema presents a significant challenge. This clinical condition ranges from minor facial edema to life-threatening swelling of vital structures. Its multifactorial etiology involves various factors and mechanisms, including C1 esterase inhibitor deficiency, food allergen hypersensitivity, and adverse drug reactions, notably involving angiotensin-converting enzyme (ACE) inhibitors and mechanistic target of rapamycin inhibitors (mTOR-Is). We present a rare case of sirolimus potentiated angioedema in a patient with long-standing ACE inhibitor therapy.

**Case:**

A 52-year-old male with a history of heart transplant developed severe upper and lower lip edema. The patient had been on Lisinopril without any adverse events. However, sirolimus was recently added to his drug regimen. Sirolimus potentiated angioedema was suspected.

**Intervention:**

Intravenous methylprednisolone, famotidine, and diphenhydramine were initiated, and both lisinopril and sirolimus were discontinued. The patient showed improvement and was discharged with oral antihistamines.

**Lessons:**

Transplant physicians should be aware of the life-threatening interaction between ACE inhibitors and mTOR-Is like sirolimus. Consideration should be given to switching from an ACE inhibitor to an angiotensin-receptor blocker when initiating patients on mTOR-Is.

## 1 Introduction

The realm of organ transplantation, particularly in the context of heart transplantation, poses a multitude of intricate challenges. Among these challenges, angioedema emerges as a significant concern and is infrequently encountered in heart transplantation. This clinical entity exhibits a spectrum of manifestations, encompassing minor and self-resolving facial edematous changes to severe and potentially life-threatening swelling that can affect critical structures such as the lips, tongue, and upper airways. The etiology of angioedema is multifactorial, characterized by a complex interplay of various contributing factors and mechanisms. These include conditions like C1 esterase inhibitor deficiency, hypersensitivity reactions triggered by specific food allergens, and adverse drug reactions, notably associated with pharmaceutical agents such as angiotensin-converting enzyme (ACE) inhibitors and mammalian target of rapamycin inhibitors (mTOR-Is) [1,2]. The latter adverse drug interaction is less known due its rare occurrence.

In this context, we present a noteworthy case that sheds light on a patient’s experience with long-standing ACE inhibitor therapy that was marked by the onset of angioedema shortly after the introduction of sirolimus into the therapeutic regimen.

## 2 Case report

**Figure 1:**
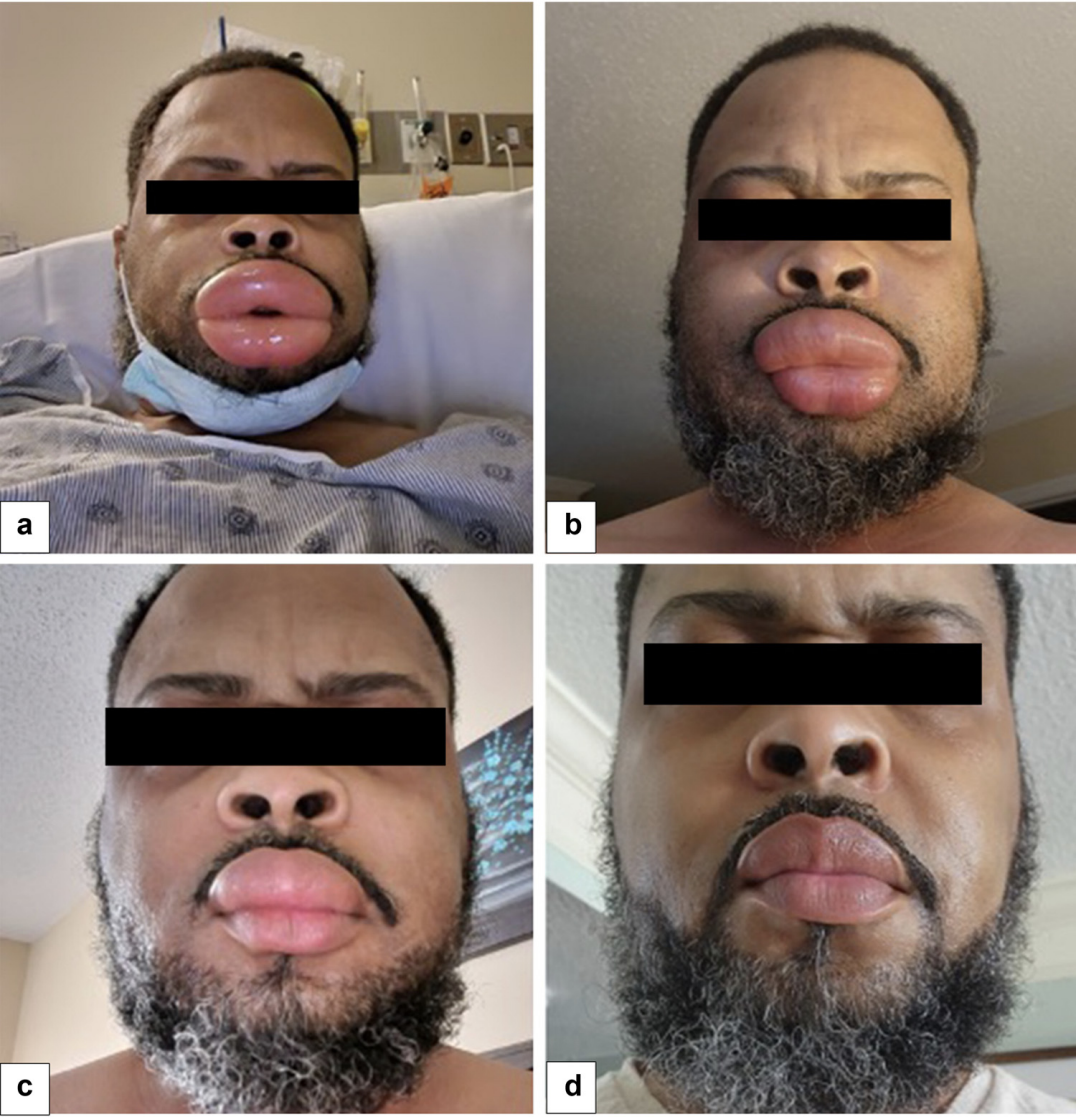
Progression and resolution of angioedema in a patient’s upper and lower lips. (a) Severe angioedema following the addition of sirolimus to the treatment regimen. Subsequently, in chronological order, panels (b)–(d) display the resolution of angioedema after discontinuation of both sirolimus and lisinopril.


A 52-year-old male, with a history of heart transplantation, presented to the emergency department (ED) with the abrupt onset of upper and lower lip edema, without concurrent respiratory symptoms. The patient had no known allergies to medications or foods and had been on a long-term daily regimen of lisinopril 5 mg. Sirolimus 1 mg daily was introduced 20 days prior as part of a renal-sparing immunosuppressive protocol. Upon ED admission, the patient’s vital signs were stable with a blood pressure of 139/83 mmHg, a pulse rate of 88 beats/min, a respiratory rate of 20, and a pulse oximetry reading of 98%. The patient exhibited no signs of difficulty swallowing. Clinical examination revealed significant edema affecting both the upper and lower lips (Figure 1a). A prompt diagnosis of angioedema was established, and treatment was initiated with intravenous methylprednisolone (125 mg), famotidine (20 mg), and diphenhydramine (50 mg). Subsequently, both lisinopril and sirolimus were discontinued. The patient’s ability to tolerate a clear liquid diet improved, and he was successfully transitioned to a soft diet. He was subsequently discharged with a prescription for oral antihistamines, demonstrating sustained improvement in the following days (Figure 1b–d).

**Informed consent:** Explicit informed consent and authorization for the use of the photographic image were obtained from the participating patient.

## 3 Discussion

Transitioning from conventional calcineurin inhibitors (CNIs) to mTOR-I, such as sirolimus, as the primary immunosuppressive regimen, is strongly advocated for cardiac transplant recipients. This transition is supported by robust evidence highlighting the effectiveness of sirolimus in preserving renal function and preventing or mitigating chronic allograft vasculopathy [3]. Clinical protocols that involve the use of sirolimus, without concurrent CNIs, particularly in renal and cardiac transplant recipients, consistently yield substantial improvements in renal function [3–9]. Despite being typically well-tolerated, mTOR-Is are linked to a spectrum of adverse events, potentially contributing to the relatively high dropout rates of 20–40% observed in clinical Phase III trials involving sirolimus and everolimus. While certain adverse effects are amenable to straightforward management, others necessitate the discontinuation of the drug [10]. Angioedema is a concerning side effect associated with the use of mTOR-Is, supported by empirical evidence derived from case series and anecdotal reports [11]. In a study by Duerr et al. involving renal transplant recipients, the incidence of angioedema was observed at 1.2% among individuals receiving mTOR-Is as part of their immunosuppressive regimen. However, when mTOR-Is were administered in combination with ACE inhibitor, the incidence significantly rose to 6.6%. Notably, patients solely on ACE inhibitor therapy exhibited angioedema in 2.2% of cases [1]. All these findings underscore the intricate interplay between ACE inhibitors, mTOR-Is, and the occurrence of angioedema.

### 3.1 ACE inhibitors and angioedema

The understanding of ACE inhibitor-induced angioedema has evolved progressively, transitioning from initial data obtained from registration trials and pharmacovigilance databases to more robust insights provided by contemporary large-scale prospective investigations. For instance, the OCTAVE trial, encompassing a vast cohort exceeding 12,000 participants, reported an incidence rate of angioedema amounting to 0.68% among individuals undergoing ACE inhibitor therapy [11]. Similarly, the ONTARGET study documented an overall occurrence rate of ACE inhibitor related angioedema at 0.3% among a population of 8,576 subjects [12]. In stark contrast, the utilization of angiotensin-receptor blockers (ARBs) was linked to a significantly lower risk of angioedema, manifesting in a mere 0.1% (*n* = 10 of 8,542 patients) incidence rate within the same trial [12]. This observed incidence concurs with recent findings from a substantial 2007 study involving 134,945 individuals, where the incidence of angioedema among patients prescribed ACE inhibitors was also reported as 0.7% [13].

### 3.2 mTOR-Is potentiated angioedema in organ transplantation

Emerging anecdotal cases have brought attention to the intricate interaction between mTOR-Is and ACE inhibitors, leading to the development of angioedema. Notably, Stallone et al. pioneered the elucidation of this phenomenon by reporting angioedema in 5 out of 52 kidney transplant recipients. These individuals experienced life-threatening tongue angioedema while concurrently being treated with a daily regimen of 5 mg sirolimus and 5 mg ramipril. Intriguingly, the onset of angioedema occurred approximately 1 month after initiating ramipril, despite prior uneventful usage of ramipril in the absence of sirolimus. It is noteworthy that all five patients exhibited relatively elevated sirolimus blood levels ranging from 16 to 20 ng/mL. The complete resolution of angioedema transpired following the discontinuation of ramipril. Subsequently, ramipril was reintroduced, albeit at reduced doses of 2.5 mg, for all patients. This was meticulously executed while maintaining sirolimus levels within the range of 8–12 ng/mL. Remarkably, this re-administration was devoid of any adverse effects, suggesting a potential dose-dependent influence of both drugs on the development of angioedema [14]. In a notable parallel observation, Fuchs and colleagues documented the occurrence of angioedema in seven heart transplant recipients within a range of 4–41 days following the initiation of everolimus treatment. It is imperative to underscore that all these patients were concomitantly undergoing combination therapy with ACE inhibitors and displayed elevated everolimus levels at the onset of angioedema. The discontinuation of ACE inhibitors and reduction of everolimus levels, maintaining them within a range of 3–8 ng/mL culminated in the complete resolution of angioedema symptoms in six out of seven patients [15]. Additionally, a randomized study involving heart transplant recipients demonstrated that those receiving ACE inhibitors and mTOR inhibitors had a 50–100 times higher incidence of angioedema compared to those on conventional immunosuppression therapy [16]. In recognition of the elevated risk of angioedema associated with the concomitant administration of ACE inhibitors and mTOR-Is, including sirolimus and everolimus, the FDA issued a warning, underlining the critical importance of these findings [17].

### 3.3 Mechanisms underlying angioedema in ACE inhibitor and mTOR inhibitor combination therapy

**Figure 2:**
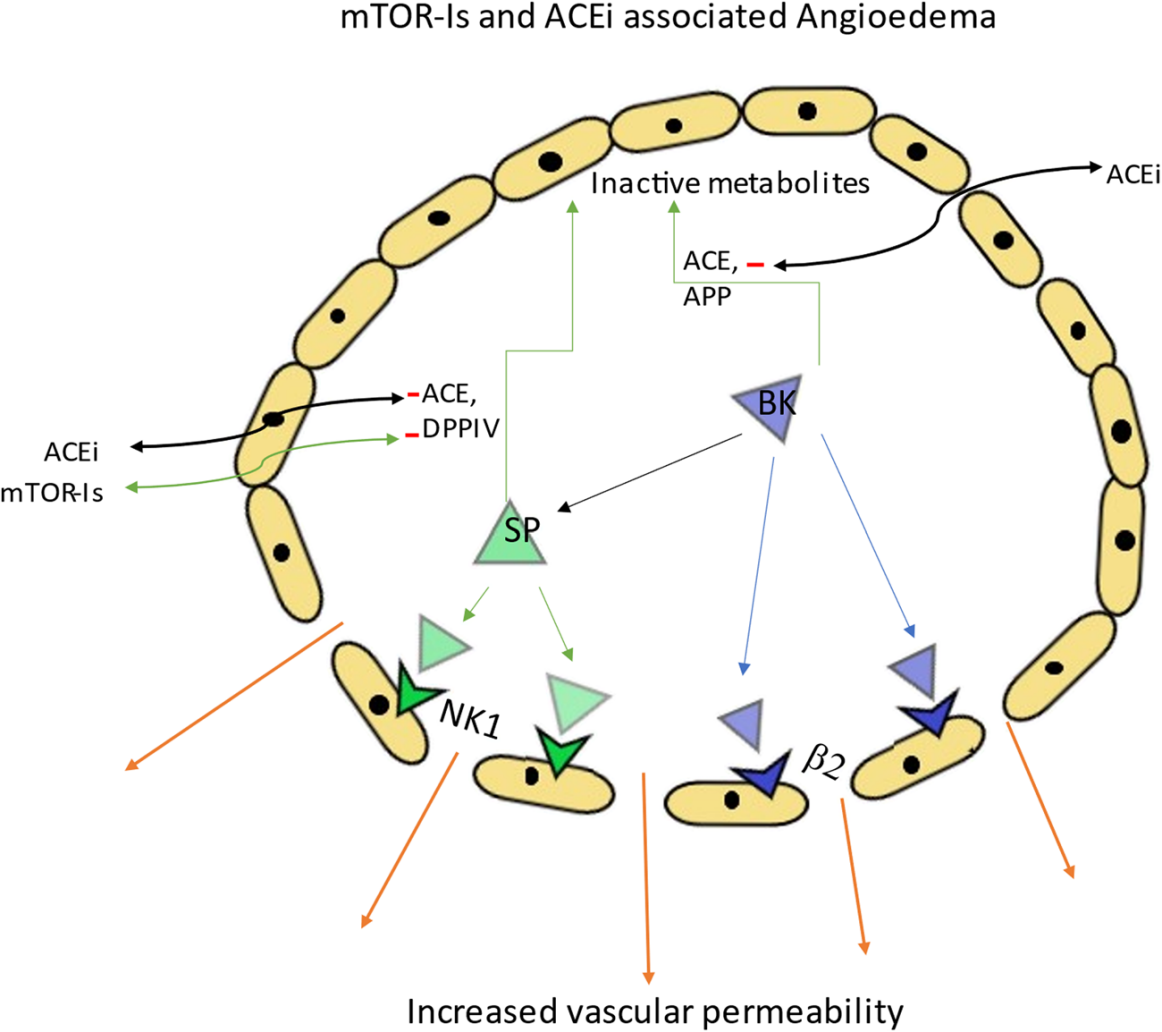
Mechanism of bradykinin (BK)-induced vascular permeability involves direct activation of its β_2_ receptor and indirect activation of NK1 receptors by stimulating the release of substance P (SP) from nerve terminals. SP then promotes vascular permeability through the activation NK1 receptors. BK and SP are typically degraded by ACE. However, during ACE inhibition, aminopeptidase P (APP) primarily degrades BK, while (DPP-IV) degrades SP.

Bradykinin and substance P are vasoactive peptides implicated in the pathogenesis of angioedema. Bradykinin primarily acts on the postcapillary venules, leading to vasodilation and increased vascular permeability through the production of nitric oxide and prostaglandins [18]. Its effect is mediated through direct stimulation of the β_2_ receptor and indirect stimulation of substance P release from nerve terminals. Substance P, in turn, activates the neurokinin 1 (NK1) receptor, further enhancing vascular permeability. Under normal conditions, ACE is responsible for degrading bradykinin and substance P. However, when ACE activity is inhibited, aminopeptidase P (APP) takes over and inactivates bradykinin, while dipeptidyl peptidase-IV (DPP-IV) inactivates substance P. Inhibition of bradykinin and substance P degradation can lead to angioedema by causing plasma extravasation into the submucosal tissue (Figure 2) [19].

DPP-IV is a cell-surface marker found on T lymphocytes involved in stimulating lymphocyte activation and proliferation [20]. Studies by Byrd et al. have shown that immunosuppressive agents, including the mTOR-I sirolimus, have the ability to reduce the expression of DPP-IV on cell surfaces and decrease its activity in circulation. Interestingly, different immunosuppressive agents inhibit DPP-IV expression through distinct mechanisms, with sirolimus exhibiting greater potency compared to conventional CNIs. This reduction in DPP-IV levels induced by sirolimus in heart transplant recipients can contribute to the development of angioedema, and the severity may be dose-related [21]. Furthermore, the use of the pharmacological DPP-IV inhibitor vildagliptin has been associated with an increased risk of angioedema in patients treated with ACE inhibitors [22]. In animal studies, genetically deficient rats lacking DPP-IV exhibited an augmented peritracheal edema response following ACE inhibitor administration, which was attenuated by a substance P receptor antagonist [23].

### 3.4 Impact on practice

Our case highlights a significant and potentially dangerous aspect of combining mTOR-Is and ACE inhibitors in therapy. This combination, commonly used in patients experiencing graft function deterioration and proteinuria, significantly elevates the risk of angioedema. Healthcare providers and patients must exercise heightened vigilance regarding this substantial interaction. Caution should be exercised when initiating either ACE inhibitors or mTOR-Is in patients concurrently prescribed the other agent. Given the current state of evidence, considering the preferential use of ARBs in patients on mTOR-Is is a prudent choice for enhanced safety [24,25]. It is important to note that advocating for the safety of everolimus over sirolimus, or vice versa, is not supported by our findings. Furthermore, we strongly recommend that transplant centers incorporate electronic medical record alerts to prevent inadvertent co-prescription of ACE inhibitors and mTOR-Is. Our overarching goal remains the reinforcement of patient safety and the continuous refinement of immunosuppressive strategies within the realm of transplantation medicine.
